# Accuracy of Ultrasound Estimation of Fetal Weight (EFW) at Term: Insights From a Malaysian Tertiary Hospital for Primary Care Implementation

**DOI:** 10.1155/ogi/9992291

**Published:** 2026-06-15

**Authors:** Woon Teen Sia, Rachel Phei Fern Yew, Prateeba Arumugam, J. Ravichandran R. Jeganathan

**Affiliations:** ^1^ Monash Medical University, Clinical School Johor Bahru, Johor Bahru, Johor, Malaysia, monash.edu.my; ^2^ Hospital Sultanah Aminah, Johor Bahru, Johor, Malaysia, moh.gov.my

**Keywords:** antenatal care, labor, prenatal ultrasound

## Abstract

**Background:**

Estimated fetal weight (EFW) is a key determinant of obstetrical decision‐making and an independent risk factor of high perinatal morbidity and mortality. However, routine ultrasound during pregnancy test is not universally available in the Malaysia primary care settings.

**Objective:**

To compare the accuracy of ultrasonographic EFW to the actual birth weight (ABW). Study DesignThis was a prospective cohort study involving women with singleton pregnancies, at term (37–38 + 6 weeks) and age ≥ 18 years. The accuracy of EFW was evaluated by calculating the percentage error (PE). PE of < 10% was regarded as “accurate.”

**Results:**

A total of 153 participants were recruited. The majority of the participants were Malay (77.8%), followed by Indian (12.4%), Chinese (4.6%), and others. There was no significant association between ethnicity and ultrasound accuracy. The mean maternal weight at booking was 64.10 kg, while the mean body mass index (BMI) was 26.09 kg/m^2^. The mean EFW was 2992.72 ± 338 g, while the mean ABW was 2960 ± 400 g. For ultrasound accuracy, the mean PE was 8.11%, with 68.63% of accurate EFW. Maternal weight, parity, and gestational diabetes were significantly associated with the accuracy of ultrasound EFW. The mean number of days from ultrasound to birth was 2.64 days. Performing an EFW within 24 h prior to delivery yielded optimal accuracy of EFW. There was a significant association between experiences of examiners with ultrasound accuracy.

**Conclusion:**

The accuracy of EFW in our study was comparable to similar studies done in other countries. There was a strong positive correlation between ultrasound EFW using the Hadlock‐4 weight estimation model with ABW in our population. The accuracy of EFW within 24 h prior to delivery warranted a calculated decision on the induction of labor. Adequate training of examiners was crucial. Routine ultrasound EFW services should be made available and accessible in all healthcare settings in Malaysia.

## 1. Introduction

Estimated fetal weight (EFW) is a key determinant of obstetrical decision‐making regarding the mode of delivery, timing for induction of labor, risk‐stratifying pregnancies, and the mapping of intrauterine growth parameters [1–3]. Furthermore, EFW is also an independent risk factor of high perinatal morbidity and mortality [2]. The World Health Organization (WHO) had recommended an ultrasound scan as a part of routine antenatal care in 2016 [4].

The two most common methods used to estimate fetal weight are ultrasound and clinical examination. The ultrasound method, when accessible, is recommended due to its better precision, objectivity, and handiness [5]. Hadlock’s formula is the most commonly used formula, which involves the measurement of biparietal diameter (BPD), head circumference (HC), abdominal circumference (AC), and femur length (FL) [1, 6]. However, the measurement variations of up to ±6–11% at or near term were reported, regardless of the application of the regression equation [7, 8]. Some studies also showed intra‐ and interobserver variabilities in determining the fetal weight via ultrasound, with the influences of several maternal and fetal factors [9–11].

The differences in the growth of EFW were significant between countries, and the Asian populations were often underrepresented in large cross‐national studies [9, 12]. The challenges of getting accurate EFW in a diverse population and the scarcity of local data were the two important drives of this study.

We examined the ultrasonography accuracy by comparing the differences of EFW with the actual birth weight (ABW). This study aimed to provide a comprehensive insight of the ultrasound EFW accuracy in the Malaysian context. Currently in Malaysia, ultrasound during pregnancy is not universally available as a routine test in the primary care settings [13]. It is hoped that this population‐based study would help inform the current healthcare policy, making ultrasound services reliable and accessible to our patients.

## 2. Materials and Methods

### 2.1. Study Population

This was a prospective cohort study carried out in the Department of Obstetrics and Gynecology of Hospital Sultanah Aminah Johor Bahru, a Malaysia tertiary hospital from December 1, 2023, to May 31, 2024. Women with singleton pregnancies in cephalic presentation, at term (37–38 + 6 weeks) and age ≥ 18 years, were included. Women with multiple pregnancies, previous scars, and fetal anomalies were excluded. The power estimate required a minimum of 129 participants to achieve a representative sample of 95% confidence level and 0.05 margin of error.

After obtaining the informed consent from the participants, the ultrasound was performed with the appointed machines, using the Hadlock‐4 formula. All participants were followed up until their deliveries to obtain the ABW of the newborns.

The discrepancy between EFW and ABW was calculated and reported as percentage error (PE).

(1) Percentage error  PE=EFW−ABWABW×100.

EFW with PE of < 10% was considered “accurate,” as the literature claimed that variations of < 10% would not affects clinical decision‐making [9, 14]. The participants were categorized and compared based on the EFW accuracy (i.e., accurate = PE < 10%; nonaccurate = PE ≥ 10%)

The examiners were grouped by their experiences in the profession (i.e., maternal–fetal medicine [MFM] specialists and obstetricians in a group, while postgraduate trainees and medical officers [MO] in the other).

In Malaysia, there was no mandatory requirement for an ultrasound to be performed at term, and pregnant women would not necessarily present for point‐of‐care assessment at the exact 37 weeks. With the average delivery in Malaysia being approximately at 39 weeks, we justified the recruitment window of 37–38 + 6 weeks of gestation, a period likely to reflect the most consistent maternal presentations prior to delivery [15].

### 2.2. Statistical Analysis

Data were analyzed using Statistical Package for Social Sciences (SPSS, Version 26). Categorical variables were presented as frequency (N) and percentage (%), while continuous variables were presented as mean and standard deviation (SD). The variables were compared and analyzed with chi‐square test (relative risk [RR] and confidence interval [CI]) and *t* test. A *p* value of < 0.05 was statistically significant for all the comparisons.

Ethical approval was granted by the Medical Research and Ethics Committee (MREC) Malaysia, NMRR ID‐23‐02927‐NEL.

## 3. Results

### 3.1. Demographic Profile

A total of 153 participants were recruited for the study. The mean maternal age was 28.6 years. Majority of them were Malay (77.8%), followed by Indian (12.4%), and Chinese (4.6%). The mean maternal height was 155.59 cm, and three (2%) had short statue (i.e., < 145 cm). The mean maternal weight at booking was 63.42 kg. The mean body mass index (BMI) at booking was 26.09 kg/m^2^. Based on the WHO Asian BMI classification, 64 (41.8%) of the participants were classified as obese, followed by normal BMI (25.5%) and overweight (21.6%). [16]. The maternal weight at booking significantly affected the EFW accuracy (*p* = 0.045). The mean maternal weight was higher in the accurate EFW group than the nonaccurate group. Further stratification by BMI categories demonstrated a consistent trend in both normal and obese groups, but not in the overweight group (Table 1).

**TABLE 1:** Demographic profile of participants.

	**All cases**	**Accurate**	**Nonaccurate**	**RR (CI)**	**p** **value**
	**(*N* = 153)**	**(*N* = 105)**	**(*N* = 48)**		

*Ethnicity (Malaysian)*					
Malay	119 (77.8)	82 (78.1)	37 (77.1)	—	0.740
Chinese	7 (4.6)	4 (3.8)	3 (6.3)		
Indian	19 (12.4)	13 (12.4)	6 (12.5)		
Others	8 (5.3)	6 (5.8)	2 (4.2)		
Age (years)	28.60 (5.36)	28.73 (5.20)	28.31 (5.74)	—	0.654
Advanced maternal age	14 (9.2)	9 (8.6)	5 (10.4)	0.931 (0.620–1.397)	0.765
Height (cm)	155.59 (5.87)	155.97 (5.42)	154.75 (6.73)	—	0.235
Short maternal statue	3 (2)	1 (1)	2 (4.2)	0.481 (0.097–2.392)	0.232
Weight at booking (kg)	63.42 (16.812) Range: 33–122.8	65.25 (17.83)	59.39 (13.67)	—	0.045^∗^

*Weight based on BMI category*					
Underweight (< 18.5) (*N* = 16) (kg)	39.12 (4.23)	37.8 (3.55)	40.81 ((4.69))	—	0.164
Normal (18.5–22.9) (*N* = 39) (kg)	51.32 (6.49)	52.92 (6.95)	47.7 (3.75)		0.018^∗^
Overweight (23–24.9) (*N* = 33) (kg)	60.39 (5.64)	59.52 (4.76)	62.71 (7.35)		0.153
Obese (≥ 25) (*N* = 64) (kg)	78.45 (12.71)	81.64 (13.4)	71.42 (7.32)		< 0.001^∗^
Morbidly obese (≥ 40) (*N* = 1)	61.6	61.60	—		—
Body mass index (BMI) at booking (kg/m2)	26.09 (6.63)	26.67 (6.99)	24.85 (5.64)		0.117

*Parity*					
Primigravida	49 (32)	28 (26.7)	21 (43.8)	0.772 (0.590–1.009)	0.036^∗^
Pseudo‐primid	6 (3.9)	6 (5.7)	0 (0)	1.485 (1.327–1.662)	0.178
Grand multipara	9 (5.9)	7 (5.7)	2 (4.2)	1.143 (0.792–1.263)	0.721
Last childbirth (LCB) < 2 years (i.e., short interpregnancy interval)	16 (10.5)	12 (11.4)	4 (8.3)	1.105 (0.814–1.500)	0.777
Last childbirth (LCB) > 5 years (i.e., long interpregnancy interval)	17 (11.1)	11 (10.5)	6 (12.5)	0.936 (0.648–1.353)	0.783
Late booker	5 (3.3)	4 (3.8)	1 (2.1)	1.172 (0.746–1.842)	1.000
Unsure of the date	10 (6.5)	5 (4.8)	5 (10.4)	0.715 (0.381–1.341)	0.288

*Note:* Continuous variables were presented as mean (SD).

Abbreviations: CI = Confidence interval, RR = relative risk.

^∗^ *p* value < 0.05. Categorical variables were presented as *N* (%).

For parity, 49 (32%) were primigravida, 6 (3.9%) were pseudo‐primid, and 9 (5.9%) were grand multipara. Being a primigravida was more likely to have an accurate EFW (RR = 1.296, *p* = 0.036). Six (10.5%) had last childbirth in < 2 years, while 17 (11%) had last childbirth > 5 years. A small proportion of participants were late booker (3.3%) and unsure of the date (6.5%) (Table 1).

Thirty‐two (20.9%) of participants had gestational diabetes mellitus. Four (2.6%) had hypertension in the current pregnancy, but none had the complications of pre‐eclampsia and eclampsia. Participants with gestational diabetes mellitus were less likely to have an accurate EFW (*p* = 0.031). The mean hemoglobin level at booking was 12.16 g/L, and 33 (21.6%) of participants had hemoglobin < 11 g/L at booking. There was only one fetus large for gestational age (LGA), one fetal growth restriction (FGR), and one case of oligohydramnios. For obstetric history, 11 (7.2%) of participants had a history of low‐birth‐weight baby (i.e., < 2.5 kg), 2 (7.2%) had a macrosomic baby, while 5 (3.3%) had experienced perinatal death in their previous pregnancy (Table 2).

**TABLE 2:** Clinical and obstetric history.

	**All cases (*N* = 153)**	**Accurate** **(*N* = 105)**	**Nonaccurate** **(*N* = 48)**	**RR (CI)**	**p** **value**

Gestational DM in current pregnancy	32 (20.9)	27 (25.2)	5 (10.4)	1.309 (1.072–1.598)	0.033^∗^
Hypertension in current Pregnancy	4 (2.6)	2 (1.9)	2 (4.2)	0.723 (0.270–1.938)	0.590
Hemoglobin (Hb) at booking (g/L)	12.16 (1.30)	12.24 (1.35)	12.00 (1.16)	—	0.301
Hb < 11 g/L at booking	33 (21.6)	20 (19)	13 (27.1)	0.855 (0.635–1.153)	0.293
Large for gestational age (LGA) in current pregnancy	1 (0.7)	1 (1)	0 (0)	1.462 (1.312–1.628)	1.000
Fetal growth restriction in (FGR) in current pregnancy	1 (0.7)	1 (1)	0 (0)	1.462 (1.312–1.628)	1.000
Oligohydramnios in current pregnancy	1 (0.7)	1 (1)	0 (0)	1.462 (1.312–1.628)	1.000
History of low birth weight (LBW) < 2.5 kg	11 (7.2)	9 (8.6)	2 (4.2)	1.210 (0.896–1.635)	0.504
History of macrosomic baby	2 (7.2)	1 (1)	1 (2.1)	0.726 (0.181–2.915)	0.530
History of gestational DM	14 (9.2)	12 (11.4)	2 (4.2)	1.280 (1.004–1.635)	0.227

*Note:* Continuous variables were presented as mean (SD).

Abbreviations: CI = Confidence interval, RR = relative risk.

^∗^ *p* value < 0.05. Categorical variables were presented as *N* (%).

Seventy‐one (46.4%) of newborns were female, while 82 (53.6%) were male. Gender was associated significantly with EFW accuracy. Conceiving a female was less likely to get an accurate EFW (*p* = 0.045). The mean gestational age when ultrasound was done was 37.91 weeks, while the mean gestational age at birth was 38.27 weeks. The mean number of days between the date of ultrasound and the actual birth date was 2.64 days. Smaller number of days was associated significantly with accurate ultrasound EFW (*p* = 0.039).

Further analysis showed a significant lower mean PE when ultrasound EFW was performed within 24 h before delivery based on our cohort (Table 3).

**TABLE 3:** Comparison of EFW discrepancies and percentage error (PE) based on the timing of ultrasound before delivery.

Timing of Ultrasound	Number of patients	Mean difference (SD) (gram)	*p* value	Mean percentage error (SD) (%)	p value
≤ 24 h vs > 24 h	80 vs 73	200.28 (150.88) vs 270.00 (194.57)	0.014^∗^	7.01 (5.61) vs 9.32 (7.09)	0.026^∗^

^∗^ *p* value < 0.05.

**FIGURE 1:**
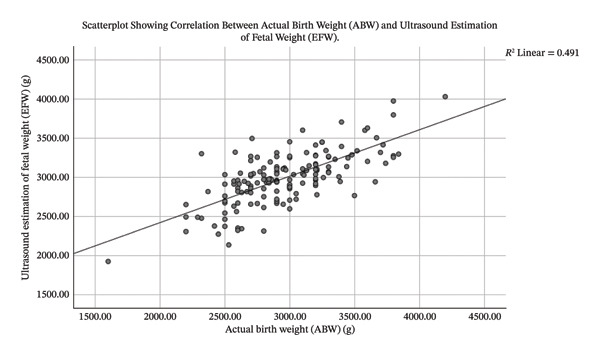
Scatterplot showing correlation between ABW and ultrasound EFW. (*r* = 0.700).

The mean ultrasound EFW was 2992.72 ± 338 g, while the mean ABW was 2960 ± 400 g (1600–4200 g) (Table 4). The mean absolute difference between the ultrasound EFW and ABW was 233.54 g, while the mean absolute PE was 8.1087%. There was a strong positive correlation (*r* = 0.700) between the ultrasound EFW and ABW (*p* < 0.001). (Figure 1).

**TABLE 4:** Details of ultrasound and babies at birth.

		**All cases (*N* = 153)**			
	**All cases (*N* = 153)**	**Accurate (*N* = 105)**	**Nonaccurate (*N* = 48)**	**RR (CI)**	**p** **value**

*Gender*					
Female	71 (46.4)	43 (41)	28 (58.3)	0.801 (0.640–1.002)	0.045^∗^
Male	82 (53.6)	62 (59)	20 (41.7)		
Week of pregnancy when US was done	37.91 (0.56)	37.92 (0.55)	37.88 (0.57)	—	0.637
Gestational age at birth (weeks)	38.27 (0.73)	38.24 (0.70)	38.32 (0.81)	—	0.510
Number of days between the date of ultrasound with actual birth date (days)	2.64 (3.81)	2.13 (3.18)	3.73 (4.77)	—	0.039^∗^
Estimated fetal weight (EFW) (*g*)	2992.73 (338.03)	3000.57 (331.40)	2975.58 (355.05)	—	0.673
Actual birth weight (ABW) (kg)	2960.92 (400.04)	3003.33 (349.45)	2868.13 (484.27)	—	0.087

*ABW based on maternal BMI category*					
Underweight (< 18.5) (*N* = 16) (kg)	2620.00 (413.67)	2717.78 (268.55)	2494.29 (546.68)	—	0.299
Normal (18.5‐22.9) (*N* = 39) (kg)	2863.85 (329.66)	2908.52 (317.90)	2763.33 (347.34)	—	0.209
Overweight (23‐24.9) (*N* = 33) (kg)	2958.18 (356.13)	2975.83 (320.08)	2911.11 (457.53)	—	0.649
Obese (≥ 25) (*N* = 64) (kg)	3113.91 (391.01)	3146.36 (340.69)	3042.50 (486.26)	—	0.395
Morbidly obese (≥ 40) (*N* = 1)	2500	2500	—	—	—

*Note:* Continuous variables were presented as mean (SD).

Abbreviations: CI = Confidence interval, RR = relative risk.

^∗^ *p* value < 0.05. Categorical variables were presented as *N* (%).

The newborns were subdivided into two weight categories. The threshold of 2900 g was preselected based on the local data and the expected distribution trends of birthweight in Malaysia [17, 18]. Further analysis revealed a significant association between newborns > 2900 g and the accuracy of EFW (*p* = 0.012). (Table 5).

**TABLE 5:** Correlation between ABW (grouped by median) and accuracy of EFW.

	**All cases (*N* = 153)**	**Accurate** **(*N* = 105)**	**Nonaccurate** **(*N* = 48)**	**RR (CI)**	**p** **value**

> 2900 g	74 (48.37)	58 (78.4)	16 (21.6)	1.317 (1.060–1.638)	0.012^∗^
≤ 2900 g	79 (51.63)	47 (59.5)	32 (40.5)		

^∗^ *p* value < 0.05.

Ten(6.5%) babies had a low birth weight (< 2500 g), and one baby was macrosomic (0.65%). At 10% margin of error, 105 (68.6%) of the newborns had accurate EFW. Among them, five (4.8%) had low birthweight, majority (94.3%) had normal weight, while one (0.9%) was macrosomic. Forty (31.4%) of the newborns had inaccurate estimation. Among them, all five babies who had low birthweight were overestimated. Twenty‐five (58.1%) of babies with normal weight were overestimated, while 18 (41.9%) were underestimated. No macrosomic baby was over‐ or underestimated (Table 6).

**TABLE 6:** Comparison of the accuracy of ultrasound EFW for babies in different weight categories.

	**Total** **(*N* = 153) (%)**	**Low birth weight** **(*n* = 10) (%)**	**Normal** **(*n* = 142) (%)**	**Macrosomic (*n* = 1) (%)**

Accurate estimation	105 (68.6)	5 (50)	99 (69.7)	1 (100)
Inaccurate estimation	48 (31.4)	5 (50)	43 (30.3)	0 (0)
Underestimation	18 (11.8)	0 (0)	18 (12.7)	—
Overestimation	30 (19.6)	5 (50)	25 (17.6)	—

Majority (87.6%) of the ultrasound were performed by postgraduate trainees and MOs, while 5.9% were by MFM specialists and obstetricians. The difference in accuracy based on the experiences of examiners was small but statistically significant (*p* = 0.022). 78% of EFW performed by MFM specialists and obstetricians were accurate, while 70% of cases performed by postgraduate trainees and MOs were accurate.

## 4. Discussion

An accurate EFW is crucial in the management of pregnancy, enabling counseling, planning, and decision‐making regarding the timing and mode of delivery [19]. In this study, the authors acknowledged and accepted the inherent error in ultrasound machine estimation up to 10% [20].

The mean PE in our study was 8.1%. This was similar to the 8.6% reported in a study done in France while lower than the 9.0% and 12.8% reported in studies done in South Nigeria and India, respectively [21–23]. 68% of newborns in our study had an accurate ultrasound EFW (i.e., 10% margin of error). This was lower than the 72% reported by Njoku et al. in South Africa and Okafor et al. in Southeast Nigeria, while higher than the 60.6% reported by Predanic et al. in New York, USA [8, 23, 24]. The mean PE and overall accuracy of ultrasound EFW in our study were comparable to studies done in different regions across the globe.

Our study showed a significant association between maternal weight at booking and ultrasound EFW accuracy. Further analysis suggested that higher maternal weight was associated with higher EFW accuracy in both normal‐weight and obese women. This was contrary to the prevailing assumption that increased maternal weight negatively affected the EFW accuracy [25–27]. One potential factor to consider was that women with normal and obese BMI might have larger fetuses [28]. A similar trend was observed in our study. Larger fetuses might improve the precision of biometric estimations. Interestingly, the comparatively lower EFW accuracy observed among overweight participants relative to obese participants was unexpected. This finding might reflect differences in sample distribution across BMI categories, statistical variation, or abdominal wall characteristics not fully captured by BMI classification alone. Further investigations incorporating sonographic parameters (e.g., amniotic fluid index), abdominal wall compliance, fat, and muscle distributions were needed to provide clearer insights.

Primigravida pregnancies were associated with a lower likelihood of accurate EFW estimation compared to multigravida pregnancies (RR = 0.77, *p* = 0.036). This might be attributed to the variations in maternal abdominal wall compliance and uterine distensibility in primigravid women that may affect the acquisition of optimal fetal biometric measurements. However, based on the available literature, there was limited evidence directly linking parity to the accuracy of EFW. Nadham et al. in Bahrain and Ashrafganjooei et al. in Iran reported no association between parity and ultrasound accuracy [29, 30].

We observed that women with gestational diabetes were more likely to have an accurate EFW. This was consistent with previous studies that reported similar or greater accuracy of EFW in pregnancies complicated by diabetes compared with nondiabetic pregnancies [31–36]. A review examining the detection and management of fetal macrosomia also noted improved accuracy of EFW in diabetic and post‐term pregnancies. However, this apparent improvement had been suggested to result from the higher prevalence of macrosomia in these groups, which might make deviations between estimated and ABW less pronounced [37]. The accuracy of EFW in diabetic mothers remained controversial.

Male fetuses had higher accuracy in EFW than females in our study. This was consistent with multiple published literature studies that claimed fetal sex as the strongest predictor of accurate EFW [10, 38]. The differences of accuracy between males and females fetuses might be attributed to the differences in their intrauterine growth pattern and biometric parameters, especially AC and FL [38, 39]. This finding encouraged the use of sex‐specific weight prediction by ultrasound [40].

Our study reported that the smaller number of days between ultrasound to delivery, the more accurate the EFW. This was consistent with other studies that recognized the time interval between the ultrasound and childbirth as an essential factor affecting the accuracy of EFW [10, 41–43]. There was a significantly lower mean PE when ultrasound EFW was performed within 24 h before delivery based on our cohort. This finding resonated with a retrospective study published recently, which recommended the time interval between ultrasonographic weight estimation and delivery to be shorter than 7 days to improve accuracy [41]. This approach could be adopted in the clinical practice to enable prompt adjustment to obstetric management plans, especially in high‐risk pregnancy or in women who were unbooked and unscreened.

There was a strong positive correlation (*r* = 0.700) between the ultrasound EFW and ABW in our study. This supported the validity and reliability of using of Hadlock‐4 weight estimation model in the Malaysian populations [44]. Our study showed that ABW of > 2900 g was associated significantly with higher EFW accuracy while fetuses with low birthweight tend to be overestimated. These findings was consistent with a Nigeria study, which highlighted the higher accuracy of EFW in the normal and macrosomic birth weight categories [19]. However, further studies comparing different estimation models were necessary.

We observed a statistically significant difference in the EFW accuracy based on the experiences of examiners. The more experiences of the operators were associated with higher accuracy of EFW. This was in line with other studies, which showed a lack of experience in ultrasound contributed significantly to the risk of inaccurate EFW [19, 22]. The high proportion of scans performed by trainees (87.6%) reflected routine practice in tertiary teaching hospitals. Although this might have modestly influenced the overall observed accuracy compared to specialist‐led studies, it enhanced the external validity of our findings by representing real‐world clinical conditions. Ultrasonography was highly operator‐dependent, and this emphasized the need for adequate training and assessments to improve patient management [19].

### 4.1. Strengths and Limitations

This study has several strengths. The prospective design minimized potential bias related to variations in ultrasound machines and calculation methods, as all ultrasound examinations utilized the same EFW formula (Hadlock‐4). Nevertheless, several limitations should be acknowledged. Although no significant association between ethnicity and EFW accuracy was observed, the relatively smaller representation of non‐Malay ethnic groups may have limited the study’s power to detect subtle ethnic differences. Additionally, while the sample size was statistically adequate for the analyses performed, it remained relatively small. As the majority of participants were term pregnancies within the normal birthweight range, these findings may not be fully generalizable to high‐risk groups such as LGA or FGR fetuses. Therefore, larger multiethnic studies with broader populations, including high‐risk subgroups, are warranted to further validate these findings and better evaluate factors influencing EFW accuracy in the Malaysian population.

### 4.2. Research Implications

We acknowledged the lack of local data and representation of Asian populations in cross‐national studies. Our study showed a strong correlation between ultrasound EFW and ABW in the Malaysian population using the Hadlock‐4 weight estimation model. This would help inform the current healthcare policy, making ultrasound services reliable and accessible to our patients.

## 5. Conclusion

EFW is a critical parameter in determining the time and model of delivery. The accuracy of EFW in our study was comparable to similar studies done in different countries. There was a strong positive correlation between ultrasound EFW using the Hadlock‐4 weight estimation model with ABW in our population. Maternal weight, parity, gestational diabetes mellitus, and gender of the fetus were associated significantly with the accuracy of ultrasound EFW. Notably, performing an EFW within 24 h prior to delivery could be clinically implemented, particularly in managing high‐risk pregnancies, as this timeframe allows for calculated decisions on the induction of labor and mode of delivery. Adequate training of examiners was crucial to improve ultrasound accuracy. Our study supported the use of ultrasound in fetal weight estimation, and we hope to inform the current healthcare policy, making ultrasound services reliable and accessible in all healthcare settings.

## Funding

This paper did not receive any funding. Open access publishing facilitated by Monash University, as part of the Wiley ‐ Monash University agreement via the Council of Australasian University Librarians.

## Ethics Statement

This study was granted by the Medical Research and Ethics Committee (MREC) Malaysia, NMRR ID‐23‐02927‐NEL.

## Conflicts of Interest

The authors declare no conflicts of interest.

## Data Availability

Research data are not shared.
